# Effects of Growth Medium Variation on the Nutri-Functional Properties of Microalgae Used for the Enrichment of Ricotta

**DOI:** 10.17113/ftb.60.01.22.7105

**Published:** 2022-03

**Authors:** Sheyma Khemiri, Ines Bouchech, Nadia Berrejeb, Mondher Mejri, Issam Smaali, Nadia Khelifi

**Affiliations:** 1 University of Carthage, National Institute of Applied sciences and Technology (INSAT), Laboratory of Protein Engineering and Bioactive Molecules (LR11ES24), BP 676, 1080, Cedex, Tunis, Tunisia; 2 University of Carthage, Higher Institute of Fisheries and Aquaculture, BP 15, Errimel, 7080, Bizerte, Tunisia; 3 University of Jendouba, Higher Institute of Biotechnology of Beja, Avenue Habib Bourguiba 9000, BP 382, Beja, Tunisia

**Keywords:** *Nannochloropsis gaditana*, *Chlorella* sp., ricotta cheese, growth media, antioxidant ability, nutritional profile, sensory evaluation

## Abstract

**Research background:**

Microalgae represent an emergent sustainable source of bioactive compounds such as antioxidants, vitamins, minerals and polyunsaturated fatty acids that can ameliorate the nutritional characteristics of foods. The biochemical composition of microalgae could be modulated by varying the culture conditions to enhance the accumulation of biomolecules of interest. The aim of this work is to optimise the nutri-functional properties of two microalgae that can be used in food production.

**Experimental approach:**

*Nannochloropsis gaditana* L2 and *Chlorella* sp. SM1 were screened for growth, biochemical composition and radical scavenging activity employing four different growth media (algal, BG-11, f/2 and Conway) with different nutrient composition. The feasibility of using *Chlorella* sp. SM1 cultivated in BG-11 medium, in an under-investigated Mediterranean dairy product ricotta cheese and its effect on the sensory attributes was investigated. Additionally, *Arthrospira platensis* was used as reference in sensory analysis.

**Results and conclusions:**

Nitrate- and phosphate-rich media (BG-11 and algal) enhanced the biomass productivity. However, the highest lipid production (23.10 and 11.86 mg/(L·day) by strains SM1 and L2 respectively) and carbohydrate content (34.79 and 44.84% by SM1 and L2 respectively) were obtained with the nitrate-deficient f/2 medium. Regardless of the used medium, the lipid profile of *Chlorella* sp. SM1 and *N. gaditana* L2 remained adequate for different applications with >50% C16-18 as the main fatty acids. Significant increase in oleic acid (C18:1) content was recorded in response to nitrogen deficiency, being the highest in SM1 in f/2 medium (34%). Nitrogen deficiency was also found to enhance phenolic compound (expressed as gallic acid equivalents, 48.8 and 35.1 mg/g in SM1 and L2 respectively) and carotenoid contents (2.2 and 2.0 mg/g in SM1 and L2 respectively). Due to its interesting antioxidant potential, *Chlorella* sp. SM1 was used at different mass fractions (0.2, 1 and 1.5%) to enrich the ricotta cheese. The sample with 0.2% *Chlorella* sp. SM1 was found to give the most appreciated product.

**Novelty and scientific contribution:**

This study presents the production of an innovative ricotta cheese using *Chlorella* sp. as a functional ingredient, without altering the manufacturing procedure, while maintaining acceptable sensorial characteristics. The biochemical composition of the used strains varied depending on the culture medium composition, which enabled the accumulation of phytonutrients of interest.

## INTRODUCTION

One of the biggest changes in the modern world diet has been in the quality of the consumed food. For that, much focus has been placed on the sources of ’green biomolecules’ such as terrestrial plants and algae. They are considered as highly efficient ’biofactories’ that produce mainly primary metabolites (lipids, proteins, carbohydrates) and secondary metabolites (carotenoids, polyphenols, terpenes, *etc*.). Plant-derived secondary metabolites, including antioxidants, have been widely studied for their potential to reduce a risk of illness and enhance the strength of the defense of the human body against pathologies such as neurodegenerative and cardiovascular diseases (*1*). Antioxidants from green sources including polyphenols, vitamins and carotenoids are becoming of great importance to replace the synthetic ones such as butylated hydroxytoluene (BHT).

Microalgae are gaining considerable interest worldwide due to their unique biomass composition extremely rich in functional ingredients, especially carotenoids, vitamins, minerals, proteins and long-chain polyunsaturated fatty acids. Microalgae do not need arable land, and can be cultured massively in controlled ponds or photobioreactors, which makes them advantageous over terrestrial plants (*2*). Although most microalgae are photoautotrophic, several strains are capable of using different carbon sources to grow heterotrophically, which may improve growth performance and biomass concentrations (*3*).

Since several essential molecules must be provided through food, microalgae have been reported to represent an excellent choice for consumers who are looking for tasty foods without harmful effects (*4*). This should replace the conventional forms of bioactive phytocompounds marketed as tablets, capsules or powders (*5*, *6*).

In the last years, several researchers have incorporated microalgal biomass in conventional food recipes to improve their basic nutritional value. Furthermore, microalgal supplementation has become economically promising for the food industry considering first the low environmental impact, and secondly the fact that consumers give importance to the relationship between diet and health (*7*). The main microalgal enrichment includes products like gluten-free bread (*8*), cookies (*9*, *10*), bread (*11*), yogurt (*12*), pasta (*13*) and biscuits (*14*). This increasing application of microalgae in foods takes advantages from their diversity and variable biochemical composition influenced by the production mode and culture medium (*15*). It has been demonstrated that nutrients (macro- and micronutrients) strongly affect the biochemical composition of the microalgae (*16*). The composition of culture medium is among the main factors affecting the bioactive compound accumulation in the algal biomass (*17*). Thus, science is still trying to domesticate novel microalgal strains to enhance their growth and improve the overall biochemical composition, which is exclusively assessed at the cultivation level. The best alternative to evaluate the effect of nutrient availability on microalgal growth and biochemical composition, on laboratory scale, is variation of the culture medium formulations.

In this context, the aim of this work is to evaluate the enrichment of a traditional dairy product, ricotta cheese, with microalgae using a commercial sample of *Arthrospira platensis* (syn. *Spirulina platensis*) as reference. It was proposed to firstly optimise the antioxidant ability and the nutritional properties of two microalgae, namely *Chlorella* sp. and *Nannochloropsis gaditana,* with potential use in food production by varying the growth media, and secondly to assess their addition to the cheese to enhance the functional properties and estimate the acceptability of the new product designated ‘Ricottalgue’.

## MATERIALS AND METHODS

### Algal strains and culture conditions

The used strains were previously isolated from two different saline sites situated in the northern part of Tunisia (North Lake lagoon: 36°49'25.6"N 10°12'36.4"E, salinity: 33.8 g/L, and Monastir lagoon: 35°46'18.0"N 10°46'34.4"E, salinity: 44.4 g/L), maintained in LIP-MB laboratory (Laboratoire d'Ingénierie des Protéines et des Molécules Bioactives, Tunis, Tunisia) and identified microscopically and molecularly as *Chlorella* sp. SM1 (GenBank accession number KM401849, NCBI, Bethesda, MD, USA) and *Nannochloropsis gaditana* L2 (GenBank accession number KT932831, NCBI) (*18*, *19*). The commercial *Spirulina* sample used in the sensory assay was provided by Bio-Gatrana Laboratories (Gatrana Sidi Bouzid, Tunisia), referenced as TN BIO 001, N° SP01018 and identified as *Arthrospira platensis*.

The strains were cultured in four different media enriched with artificial seawater: algal (*20*), BG-11 (*21*), f/2 (*22*) and Conway medium (*23*). The nutrient composition of each medium is detailed in Table S1 and all the chemicals were purchased from Sigma-Aldrich S.a.r.l, Merck (Saint-Quentin Fallavier, France). All cultures were first grown in 500-mL Erlenmeyer flasks, then transferred to 2-litre glass reactors (0.07 m diameter, 0.5 m length) with the respective growth media. All experiments were conducted in batch mode and under controlled conditions of light intensity (200 μmol/(m^2^·s)) with a photoperiod 14:10 (light/dark), temperature 23 °C and continuous aeration (0.2 L/(L·min)). The absorbance was determined colorimetrically at 750 nm (spectrophotometer model 1240 UV-Vis; Shimadzu, Kyoto, Japan). Cell counts were performed every 3rd day under optical microscope (model Pégase; Nachet, Paris, France) using Malassez counting chamber (depth 0.200 mm; Hecht Assistent®, Sondheim vor der Rhön, Germany). Algal biomass concentration (*γ*/(g/L)) was measured gravimetrically after drying a centrifuged (13 000×*g* for 5 min, model MPW-350R; MPW Med. Instruments, Warsaw, Poland) culture sample at 105 °C for 24 h.



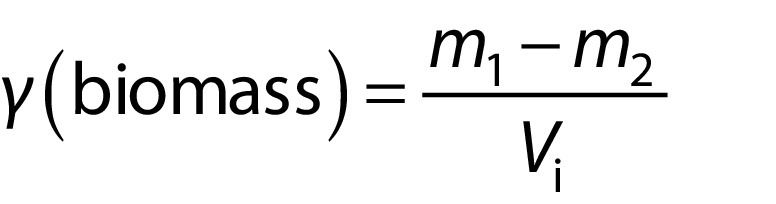



where *m*_1_ is the initial mass, *m*_2_ is the mass of the biomass after drying, and *V*_i_ is the initial volume.

Different growth parameters were determined as follows (*24*):



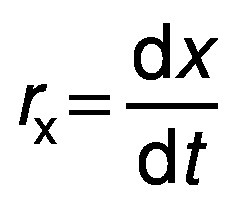



where *r*_x_ is biomass productivity, *x* is biomass concentration in mg/L and *t* is time in days.



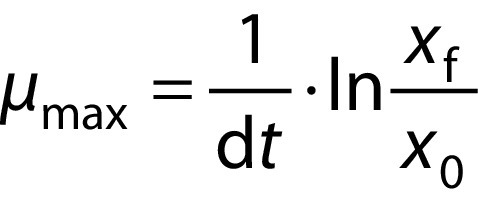



where *μ*_max_ is specific growth rate and *x*_0_ and *x*_f_ are the mean dry biomass concentrations at the times *t*_0_ and *t*_f_, respectively, and:



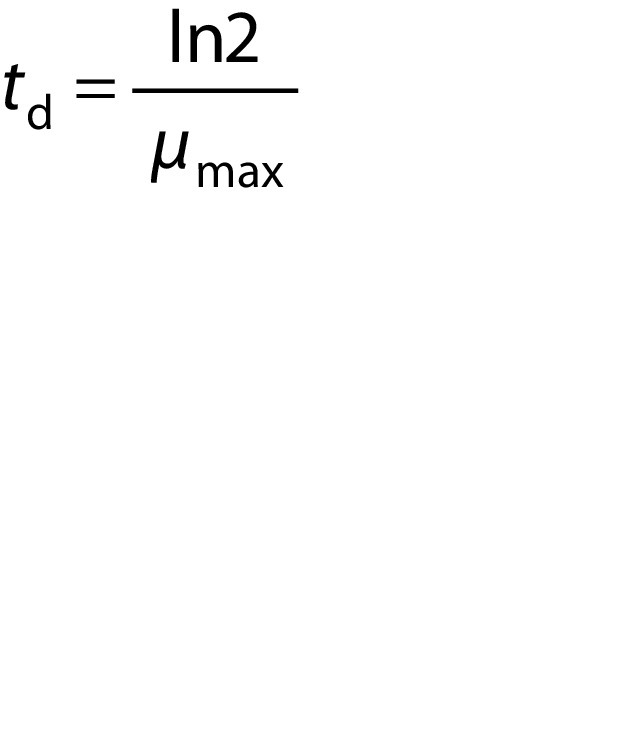



where *t*_d_ is biomass generation time.

### Preparation of microalgal extracts

The biomass was harvested at the beginning of the stationary phase of culture and washed twice by centrifugation at 13 000×*g* for 5 min (MPW-350R; MPW Med. Instruments, Warsaw, Poland). After harvesting, the microalgal biomass was dried at 60 °C in a regular oven (ED115UL; Binder GmbH, Tuttlingen, Germany) for 24 h until there was no change in the mass. Microalgal biomass was then macerated in ethanol (1:15 *m*/*V*) for 3 h at room temperature in darkness under agitation (130 rpm, Precision 2871 reciprocating water bath; Thermo Scientific, Waltham, MA, USA). The extraction was repeated several times until the supernatant was colourless. The extraction method was selected and adjusted on the basis of the results obtained by Maadane *et al.* (*25*) on different microalgal biomass, which permitted to obtain an efficient extraction of biomolecules. The extracts were filtered (membrane filters 0.2 µm; Whatman®, Merck) and the solvent was removed by rotary evaporation (IKA RV-10; IKA®-Werke GmbH & Co. KG, Staufen, Germany) at 40 °C. All concentrated extracts were weighed and stored at −20 °C until use.

### Biochemical composition

Total lipids were measured according to a modified version of Kochert’s method (*26*). Briefly, the dried biomass was ground with an equivalent mass of alumina for 5 min. A methanol/chloroform (2:1 *V*/*V*) solution was added to the biomass and alumina mixture. The mixture was then centrifuged (2054×*g* for 5 min, MPW-350R; MPW Med. Instruments). The pellet underwent three additional extractions. To the final supernatant, 158 mM HCl (Sigma-Aldrich S.a.r.l, Merck) and 0.015% MgCl_2_ (Sigma-Aldrich S.a.r.l, Merck) were added. The lower phase containing the lipid fraction was extracted using a Pasteur pipette and transferred to a new pre-weighed tube.

Lipid productivity was determined as follows:

*r*_p_=*w*(lipid)·*r*_x_ /5/

where *r*_p_ and *r*_x_ are lipid and biomass productivity.

Fatty acids were transesterified according to ISO 12966-2:2017 (*27*). The resulting fatty acid methyl ester (FAME) composition was determined by gas chromatography (CP-3800 GC; Varian, Walnut Creek, CA, USA) equipped with 30 m SUPELCOWAX 10 capillary column (0.32 mm internal diameter and 0.25 μm film thickness). The injector (split 1:50) and detector (flame ionization) temperatures were kept constant at 250 °C. The oven temperature program started at 200 °C for 8 min, then increased up to 230 °C at 5 °C/min and maintained constant at that temperature for 16 min. Helium was used as the carrier gas, and kept at a constant flow rate of 1.3 mL/min. The content of FAMEs was calculated as the mass fractions of the total fatty acids present in the sample, determined from the peak areas. The 1, 2-diheptadecanoyl-*sn*-glycero-3-phosphocholine (17:0) was used as an internal standard. Fatty acid content was calculated according to the UNE EN 14103:2020 (*28*).

Mass fractions of carbohydrates were determined using the conventional phenol-sulfuric acid method developed by DuBois *et al.* (*29*) with glucose as a standard. The sample was mixed with 72% sulfuric acid (Merck) at 30 °C for 1 h. The mixture was autoclaved (BKM-Z12N; Biobase, Jinan, Shandong, PR China) for 1 h at 120 °C after the dilution of the sulfuric acid to 4%. An equivalent volume of 5% phenol solution (Merck) and 1 mL of concentrated sulfuric acid where added quickly to the lipid fraction. Then the mixture was heated to 100 °C for 5 min. After 30 min at room temperature, the absorbance was read at 480 nm (1240 UV-Vis spectrophotometer; Shimadzu).

Relative proteins were estimated from the difference between the total dry ash-free biomass and the sum of the lipid and carbohydrate contents (*19*).

### Phenolic content

Phenolic content was determined using Folin-Ciocalteu reagent based on the slightly modified method of Singleton and Rossi (*30*), using gallic acid as a standard. Briefly, 125 µL of extracts (1 mg/mL) were mixed with an equal volume of Folin-Ciocalteu reagent (Sigma-Aldrich, Merck), 1 mL of 7% sodium carbonate and the volume was made up to 3 mL by adding distilled water. The mixture was mixed and incubated for 90 min in the dark. After that, the absorbance was measured at 760 nm (1240 UV-Vis spectrophotometer; Shimadzu) and the results were expressed in mg gallic acid equivalents (GAE) per g dry extract.

### Carotenoid content

Carotenoid content of algal extracts was estimated spectrophotometrically according to Lichtenthaler and Buschmann (*31*) method. Aliquots of the extracts were prepared at concentration of 1 mg/mL in ethanol. Absorbances were measured at 470, 648 and 664 nm (1240 UV-Vis spectrophotometer; Shimadzu), and carotenoid content was calculated using the Lichtenthaler equations (*25*, *31*).

*w*(Chla)=13.36·*A*_664 nm_-5.19·*A*_648 nm_ /6/

*w*(Chlb)=27.43·*A*_648 nm_-8.12·*A*_664 nm_ /7/

*w*(carotenoid)_total_=(1000·*A*_470 nm_-1.63·Chla-104.96·Chlb)/221 /8/

where Chla and Chlb are chlorophyll a and b respectively. The numbers in the equations are the specific absorbance coefficients.

### DPPH radical scavenging assay

The antioxidant capacity of the samples was evaluated by their ability to scavenge the DPPH (2,2-diphenyl-picryl-hydrazyl-hydrate; Sigma-Aldrich) radical at various concentrations. The method of Kumar *et al.* (*32*) was used with slight modifications. A volume of 1 mL of 0.2 mM (in absolute ethanol) DPPH solution was added to 0.2 mL of different methanolic extracts (1, 2, 3, 4 and 5 mg/mL). Absorbance was measured at 517 nm (1240 UV-Vis spectrophotometer; Shimadzu) after 30 min of incubation in the dark against methanol as blank. DPPH radical scavenging capacity (%) was calculated as follows:

Scavenging capacity=((*A*_control_-*A*_sample_)/*A*_control_)·100 /9/

where *A*_control_ is the absorbance of the control (DPPH), and *A*_sample_ is the absorbance of the tested sample (with DPPH). The absorbance of extracts in methanol (without DPPH) was determined in order to subtract the absorbance of coloured extracts.

The IC_50_ was calculated by linear regression, and expressed in milligram per millilitre. Butylated hydroxytoluene (BHT) was used as reference standard for both strains.

### Ricotta cheese production

The control ricotta cheese sample was prepared by heating whey (FarmCheese, Manouba, Tunisia) obtained from cow’s milk, which was previously coagulated with rennet, at 45 °C by adding salt and continuing heating in large open kettles until the temperature reached 80 to 85 °C. At that point, a suitable food grade acidulant (citric acid) was added to reduce the pH to 6.0 and induce coagulation of the proteins. The curd particles float to the surface of the hot liquid, are scooped off and placed in a perforated recipient. Then, to coagulated whey the microalgal biomass was added at 0.2, 1 and 1.5% mass fraction between different layers to obtain homogeneous product. The samples were left to drain and cool overnight.

### Sensory analysis

Hedonic evaluation of the ricotta supplemented with the biomass of *Chlorella* sp. SM1 obtained from BG-11 medium and commercial *Arthrospira platensis* biomass, as well as the control sample was performed based on the protocol previously described by Batista *et al.* (*9*). *Chlorella* sp. SM1 was selected for this analysis instead of *Nannochloropsis gaditana* L2 because of its interesting biochemical profile and a good biomass quantity to be used in the ricotta. Sensory evaluation was carried out in an appropriate room at 25 °C with adequate lighting respecting the international standard (ISO 8589:2007) (*33*). The main purpose of the study was clearly explained to the panellists (INSAT researchers) who had to sign an informed consent in order to express their agreement to participate in this research program. Each panel member was trained how to score different characteristics. Ricotta samples were served a day after being cooked in random order to 30 individuals, 26 female and 4 male aged between 23 and 45, who were asked to evaluate the following attributes of the samples: colour, odour, taste, texture and global appreciation (5 levels from ‘very pleasant’ to ‘very unpleasant’). Panellists were also asked whether they would buy the ricotta they tested (from ’would certainly buy’ to ’certainly would not buy’).

### Statistical analysis

The results of three replicates from each sample were used for statistical analysis and the values were expressed on dry mass basis as mean value±standard deviation. Origin Pro v. 8.0 software (*34*) was used. Results were compared among culture conditions and strains by one-way ANOVA in conjunction with Tukey’s test at a significance level of 95% (p<0.05).

## RESULTS AND DISCUSSION

### Growth performance of microalgae

**Fig. S1:**
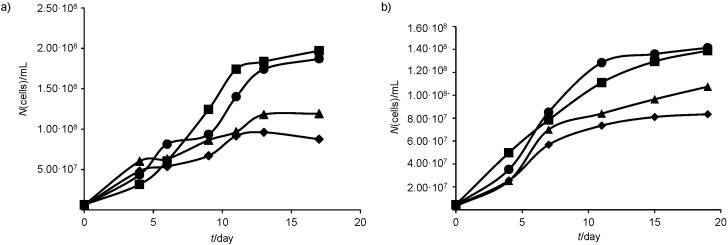
Cell growth of: a) *Chlorella* sp. SM1 and b) *Nannochloropsis gaditana* L2 cultivated in four culture media (algal (dot), BG-11 (square), f/2 (triangle) and Conway (lozenge)

Effects of different medium compositions on growth parameters of *Chlorella* sp. SM1 and *Nannochloropsis gaditana* L2 are shown in Table 1 and Fig. S1. The two strains followed conventional growth curve (lag, exponential and stationary phase). It can be observed that *Chlorella* sp. SM1 grew better in BG-11 medium, yielding 1.97·10^8^ cells/mL after 17 days. The highest number of *N. gaditana* L2 cells was obtained in the algal medium (1.42·10^8^ cell/mL). Results in Table 1 confirm that *Chlorella* sp. SM1 and *N. gaditana* L2 achieved higher growth rates and biomass productivities in BG-11 and algal media. The biomass productivity of the two strains was significantly different when growing in the same culture medium (Table 1). However, among the selected growth media, f/2 gave the highest lipid productivities. Biomass productivities found in this work were higher than those reported by George *et al.* (*35*) for *Ankistrodesmus falcatus* cultivated in BG-11 and Bold’s basal medium medium (6.14 and 1.6 mg/(L·day) respectively). Xia *et al.* (*36*) mentioned comparable biomass and lipid productivities (75.4 and 21 mg/(L·day), respectively) for *Desmodesmus* sp. cultivated in modified BG-11 medium.

**Table 1:** Specific growth rates (*μ*_max_), generation time (*t*_d_), biomass productivity (*r*_x_) and lipid productivity (*r*_p_) of *Chlorella* sp. SM1 and *Nannochloropsis gaditana* L2 cultivated in four different growth media

Medium	*μ*_max_/day^-1^	*t*_d_/day	*r_x_/* (mg/(L·day))	*r*_p_/ (mg/(L·day))	
*Chlorella* sp. SM1					
Algal	(0.33±0.01)^aA^	(2.13±0.03)^aA^	(128.9±12.7)^aA^		(13.2±0.3)^aA^
BG-11	(0.34±0.01)^aA^	(2.04±0.03)^aA^	(133.6±16.4)^aA^		(7.8±0.7)^bA^
f/2	(0.10±0.01)^bA^	(7.1±0.7)^bA^	(71.5±8.3)^bA^		(23.1±0.9)^cA^
Conway	(0.10±0.01)^bA^	(7.2±0.7)^bA^	(58.1±12.4)^bA^		(18.0±1.6)^dA^
*Nannochloropsis gaditana* L2					
Algal	(0.49±0.05)^aB^	(1.4±0.2)^aB^	(91.2±23.3)^aA^		(4.04±0.04)^aB^
BG-11	(0.47±0.05)^aB^	(1.5±0.2)^aB^	(74.0±26.4)^aB^		(4.77±0.09)^aB^
f/2	(0.22±0.05)^bB^	(3.4±0.8)^bB^	(3416±5.7)^bB^		(11.9±1.4)^bB^
Conway	(0.20±0.05)^bB^	(3.8±1.0)^bB^	(33.2±5.3)^bB^		(7.8±1.2)^cB^

Mean values and standard deviations followed by the same lower-case letter in the column did not differ significantly among culture media for each strain, and mean values followed by the same capital letter in the column did not differ significantly between the strains in the same culture medium based on Tukey’s test (p>0.05), *N*=3

**Fig. 1:**
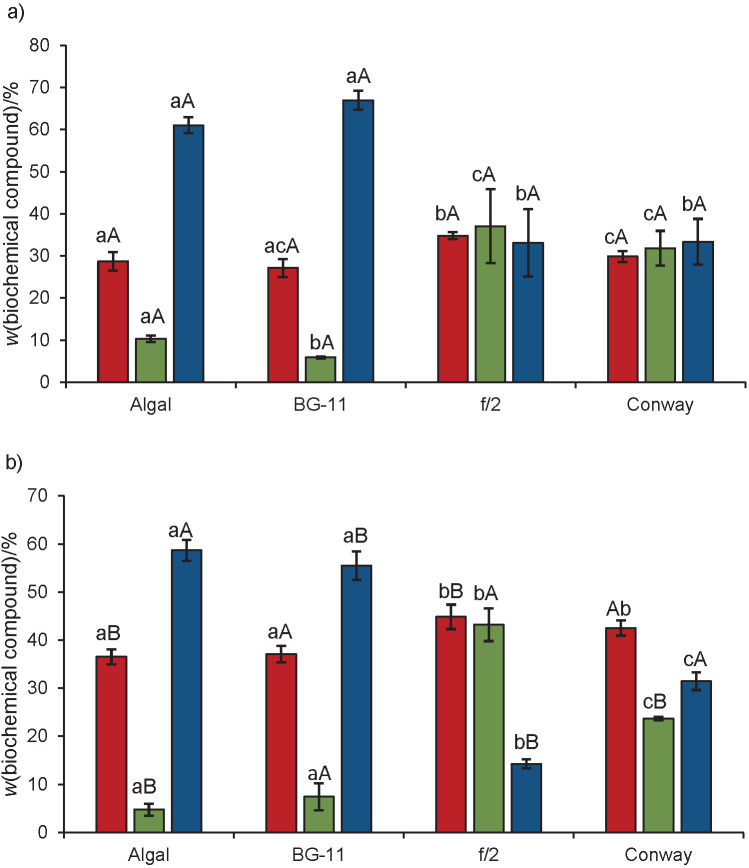
Biochemical composition of: a) *Chlorella* sp. SM1 and b) *Nannochloropsis gaditana* L2 cultivated in four culture media (algal, BG-11, f/2 and Conway). Carbohydrate (brown), lipid (green) and protein (blue). The same lower-case letters indicate a non-significant difference between the culture media for each strain, and the same capital letters indicate a non-significant difference between strains in the same culture medium based on Tukey’s test (p>0.05). Results were expressed as mean value±standard deviation (*N*=3)

The richness in macronutrients (nitrogen and phosphorous) significantly influences microalgal cultivation (Table S2). Thus, it is obvious that the presence of higher concentrations of N and P in algal and BG-11 media is important for the cell growth, while their limitation leads to poor growth ability and higher lipid productivity. These results are similar to those of Jazzar *et al.* (*19*), who suggested that N and P are important for cell division and protein accumulation. N is converted inside algal cells into a useable form, *i.e.* nitrite that is reduced to ammonium, which in turn produces glutamine responsible for protein production. This explains the lower protein contents obtained in N-deficient f/2 medium (Fig. 1). *N. gaditana* L2 reached its highest biomass productivity and cell growth in algal medium, which had the highest P concentration, fivefold higher than BG-11 medium. Indeed, excess phosphorus concentration results in excess of ATP and NADPH synthesis, carrying energy for cell functions, which in turn, enhance growth and biomass production (*37*).

Accordingly, previous studies have demonstrated that lipid and biomass production is affected by N:P ratios in culture media (*19*). The N:P ratios in f/2 and Conway media used here were 29:1 and 7:1, respectively, which seems suitable for higher lipid production, while N:P ratios 16:1 and 98:1 (algal and BG-11 media respectively) induce optimal cell growth. This was also reported by Chia *et al.* (*38*), who mentioned a higher lipid production in *Chlorella vulgaris* at N:P ratio 10:1 but not 100:1.

Micronutrients such as magnesium, sulfur and iron play a significant role in the growth of microalgae (*39*). Previous studies have reported the contribution of iron to microalgal growth as one of the most important trace metals involved in the oxidation–reduction of photosynthesis pathway. Magnesium is a constituent of chlorophyll, playing a key role in O_2_/CO_2_ utilization in photosynthesis. Sulfur is involved in cell division and lipid accumulation. Wong *et al.* (*40*) reported a lower growth rate of *Chlorella vulgaris* when cultivated under iron-deficiency coupled with lower amount of Mg and S. The lower Mg and Fe concentrations are observable in f/2 and Conway media, while S is totally absent from Conway medium.

### Biochemical characterization

#### Primary metabolites

Fig. 1 shows the impact of varying the growth media (algal, BG-11, f/2 and Conway) on *Chlorella* sp. SM1 and *Nannochloropsis gaditana* L2 biomass composition. The biochemical composition of the two strains varied significantly in response to nutrient availability in the growth media. *Chlorella* sp. SM1 accumulated the highest mass fractions of lipids and carbohydrates in f/2 medium (Fig. 1a). The same behaviour was reported for *N. gaditana* L2 (Fig. 1b). The highest mass fraction of protein was measured in *Chlorella* sp. SM1 (67.0%) cultivated in BG-11, while *N. gaditana* L2 accumulated the highest protein mass fraction in algal medium (58.7%). Proteins from microalgae are well appreciated for human consumption due to the high content of essential amino acids. Matos *et al.* (*41*) mentioned an average protein mass fraction of 40% in six different types of microalgal biomass determined by Kjeldahl method using a nitrogen-to-protein conversion factor of N×4.78. It should be noted that the estimates for the crude protein include other nitrogen compounds, which in general are expected to account for around 10% of the total nitrogen found in microalgae. The same team (*41*) registered a lower lipid (8.1 and 15.6%) and carbohydrate mass fractions (18.6 and 16.7%) in *N. gaditana* and *N. oculata* cultivated in f/2 medium. *Chlorella* sp. SM1 recorded twofold increase in lipids compared to *Chlorella vulgaris* cultivated in several growth media (*40*) and an interesting carbohydrate content compared to *Chlorella* sp. cultivated under nitrogen-limited condition (*41*).

Our findings indicated that N-deficient medium, particularly f/2, is suitable for enhancing lipid and carbohydrate accumulation, while at higher nitrate and phosphate concentrations (in algal and BG-11 media), higher protein content was obtained (*19*, *40*).

#### Carotenoids and phenolic compounds

Microalgae are known to be an interesting source of antioxidants, including skeleton carbon compounds (as carotenoids and phenolic compounds), that play an important role in scavenging reactive oxygen species generated during photosynthesis. In order to investigate the effect of nutrient availability on the accumulation of antioxidants in the studied microalgae, total carotenoid and phenolic contents of *N. gaditana* L2 and *Chlorella* sp. SM1 obtained in different culture media (algal, BG-11, f/2 and Conway) were determined. Total carotenoid and phenolic mass fractions in both strains differed significantly among the tested media (Table 2 (*15*, *42*, *43*)). Briefly, carotenoid and phenolic mass fractions were enhanced in f/2 and Conway media. The lowest antioxidant mass fraction; however, was observed in BG-11 and algal media. This suggests that both strains accumulated larger amount of antioxidants when cultivated under nitrate limitation (f/2 and Conway media). Previous works have reported the effect of modulating nitrate availability on the accumulation of carotenoids in some microalgal species such as *Dunaliella* and *Haematococcus*, well known for exploiting significant percentage of secondary metabolites under nutrient stress (*44*). Moreover, it is important to note that a limited number of papers have described the phenolic content in microalgae, especially when it is associated with nutrient stress. For a better discussion of our results, Table 2 further summarizes the comparison of carotenoid and phenolic contents reported in some *Chlorophycae* strains. Regardless of the effect of the composition of different growth media, both phenolic and carotenoid mass fractions of the analysed extracts were comparable to those reported earlier (*15*, *25*, *45*). Nevertheless, when comparing the results reported here with other studies, it should be taken into consideration that the content and composition of carotenoids and phenolic compounds are typically influenced by other stress factors such as extracting solvents (*25*), UV stress (*46*) or metal stress (*47*). Taken altogether, it would be interesting in the future to cultivate the strains under investigation in two stages. In the first stage, microalgae could be cultivated in N- and P-rich algal or BG-11 medium to ensure a good biomass productivity. In the second stage, the cells should be transferred, at the end of their exponential growth phase, to N- and P-deficient f/2 medium for better secondary metabolite accumulation.

**Table 2:** Comparison of carotenoids and phenolic mass fractions reported for different microalgal extracts under different culture conditions with the present study

Strain	Nutrient medium	Other culture conditions	*w*(carotenoids)/(mg/g)	*w*(phenolics as GAE)/(mg/g)	Reference
*Chlorella* sp. SM1					
	Algal	Air flow rate 0.2 L/min, luminosity 200 μmol/(m^2^·s), photoperiod light/dark=14:10 *t*=23 °C	(0.9±0.1)^aA^	(27.8±2.3)^aA^	Current study
	BG-11		(0.76±0.04)^aA^	(10.2±0.5)^bA^	
	f/2		(2.2±0.3)^bA^	(41.4±2.7)^cA^	
	Conway		(2.17±0.04)^bA^	(48.8±4.5)^cA^	
*Nannochloropsis gaditana* L2					
	Algal	Air flow rate 0.2 L/min, luminosity 200 μmol/(m^2^·s), photoperiod light/dark=14:10 *t*=23 °C	(0.61±0.06)^aB^	(20.8±3.5)^aB^	Current study
	BG-11		(0.76±0.05)^aA^	(15.0±1.1)^aB^	
	f/2		(12.0±0.2)^bA^	(52.7±7.6)^bA^	
	Conway		(1.6±0.4)^bB^	(35.1±3.4)^cB^	
*Chlorella vulgaris*					
	Control: *c*(N)=5 mM	Air flow rate 0.25 L/min, luminosity 125 µmol/(m^2^·s), photoperiod light/dark=12:12	3.8	3.3	(*15*)
	*c*(P)=0.25mM				
	*c*(P)_limitation_=0.01 mM		0.9	1.8	
	*c*(N)_limitation_=0.2 mM		0.4	1.3	
*Tetraselmis suecica*					
	Control: *c*(N)=5 mM	Air flow rate 0.25 L/min, luminosity 125 µmol/(m^2^·s), photoperiod light/dark=12:12	2.5	3.3	(*15*)
	*c*(P)=0.25 mM				
	*c*(P)_limitation_=0.01 mM		1.3	2.8	
	*c*(N)_limitation_=0.2 mM		0.5	1.5	
*Chlorella elipsoida*					
	Control: *c*(N)=10 mM	^Flow rate (0.03 % CO^2 in air), luminosity 919 or 1839 µmol/(m^2^·s) under *c*(N)_limitation_ *t*=(25±3) °C	6.5	0.9	(*42*)
	*c*(N)_limitation_=0.7 mM		30.4	4.31	
*Haematococcus pluvialis*					
	Control: *c*(N)=6.3 mM	Air flow rate 0.05 L/min, luminosity 850 μmol/(m^2^·s) *t*=25 °C	0.7*	n.d.	(*43*)
	*c*(N)_limitation_=1.5 mM		10*	n.d.	

Mean values and standard deviations followed by the same lower-case letter in the same column did not differ significantly among culture media for each strain, and mean values followed by the same capital letter in the same column did not differ significantly between the strains in the same culture medium based on Tukey’s test (p>0.05), *N*=3. n.d.=not determined, *astaxanthin

### DPPH radical scavenging activity

**Fig. 2:**
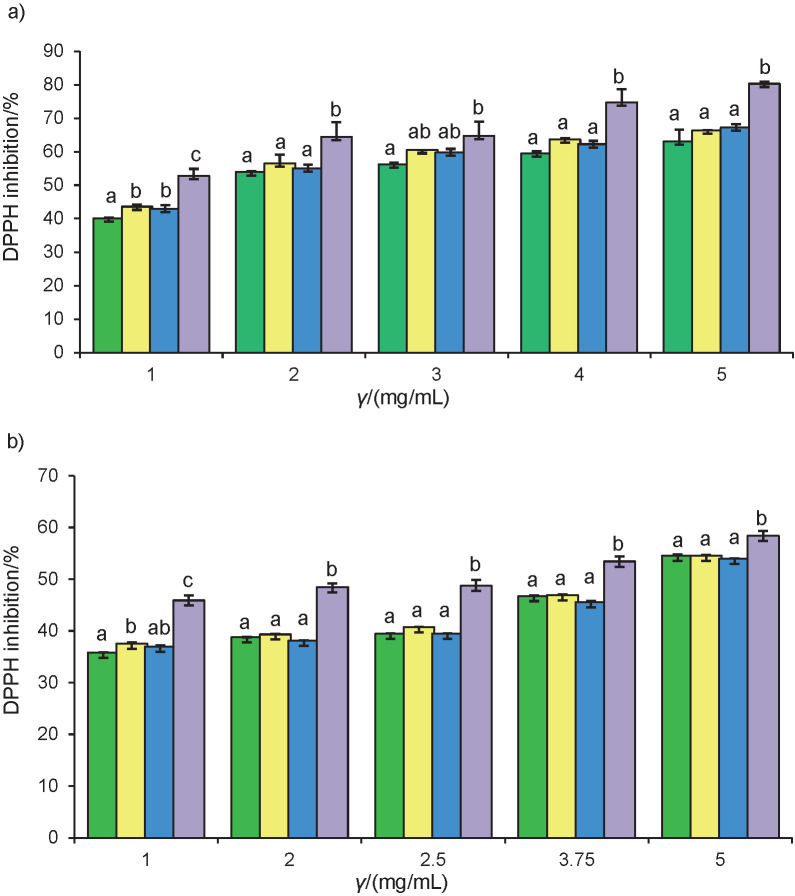
DPPH radical scavenging activities in different methanolic extracts of: a) *Chlorella* sp. SM1 and b) *Nannochloropsis gaditana* L2. BG-11 (green), algal (yellow), Conway (blue) and f/2 (purple). The same lower-case letters indicate a non-significant difference between the culture media for each strain at each concentration, based on Tukey’s test (p>0.05). Results are expressed as mean value±standard deviation (*N=3*)

The antioxidant activity of the samples was assessed using the DPPH as a stable free radical. The different inhibition capabilities of the methanolic extracts obtained from *N. gaditana* L2 and *Chlorella* sp. SM1 at different concentrations in different growth media (algal, BG-11, f/2 and Conway) are shown in Fig. 2. The IC_50_ values of the extracts were calculated and compared with the standard antioxidant BHT (Table S2).

Fig. 2 shows the ability of both strains to reduce the DPPH, which considerably increased with increasing concentration of the strains. Methanolic extract of 5 mg/mL *Chlorella* sp. SM1 in f/2 medium had the highest scavenging ability (80%), while that of *Nannochloropsis gaditana* L2 at the same concentration was 58%. Previous works emphasized an interesting DPPH radical scavenging activity of *Chlorella* species (*45*, *48*, *49*).

The IC_50_ values ranged from (4.49±0.05) to (2.5±0.3) mg/mL for *N. gaditana* L2 and from (1.97±0.07) to (1.2±0.3) mg/mL for *Chlorella* sp. SM1 (Table S2). The lowest IC_50_ values were obtained in f/2 medium. The tested extracts possessed a radical scavenging activity lower than the BHT that was used as positive control, exhibiting an IC_50_ of 0.56 mg/mL.

The antiradical activity of the extracts of both strains did not differ significantly in algal, BG-11 and Conway media. However, it was significantly higher in N-deficient f/2 medium. Thus, it is clear that when reducing the nitrate content of the medium to a moderate level (from 1.5 g/L in BG-11 medium to 0.99 and 0.074 g/L in algal and Conway media respectively), the inhibition of *Chlorella* sp. SM1 and *N. gaditana* L2 methanolic extracts remained apparently unaffected (Fig. 2). The nitrate concentration in f/2 medium presented a stressed condition for the evaluated strains, which has been translated in a significant increase in the ability of scavenging DPPH at different concentrations. Elevated antioxidant activity under low nitrate concentration was previously recorded in nine microalgal species (*50*).

### Fatty acid profiles

The quality of lipids obtained from each strain cultivated in a different medium (algal, BG-11, f/2 and Conway) was assessed by gas chromatography after transesterification of all extracts. The variation in the mass fraction of PUFA (polyunsaturated fatty acids), MUFA (monounsaturated fatty acids) and SFA (saturated fatty acids) is summarized in Table 3.

**Table 3:** Mass fractions of fatty acids in *Chlorella* sp. SM1 and *Nannochloropsis gaditana* L2 cultivated in different media

		*w*(fatty acid)/%								
Fatty acid	*Chlorella* sp. SM1						*Nannochloropsis gaditana* L2			
	Algal		BG-11	Conway	f/2		Algal	BG-11	Conway	F/2
C14:0	n.d.		0.5	0.9	0.7		n.d.	n.d.	0.8	0.8
C16:0	20.3		17.2	33.4	30.9		20.5	17.9	30.4	27.7
C16:1	0.3		n.d.	nd	1.7		4.4	3.4	2.2	2.3
C18:0	nd		n.d.	nd	nd		2.8	2.3	3.8	3.3
C18:1	7.0		17.9	29.6	34.0		4.5	3.1	26.2	24.5
C18:2	32.9		35.8	17.1	18.2		42.5	35.9	20.5	23.2
C18:3	8.6		5.7	7.7	7.9		n.d.	10.8	8.2	8.6
C20:0	2.5		1.1	0.3	0.7		n.d.	3.5	0.2	0.4
C22:1	n.d.		n.d.	0.2	0.2		n.d.	n.d.	0.2	0.2
C24:0	2.3		n.d.	0.2	nd		n.d.	n.d.	0.2	0.3
SFA	(25.1±0.4)^aA^		(18.8±0.4)^bA^	(34.8±0.5)^cA^	(32.3±0.4)^dA^		(23.3±0.3)^aA^	(23.7±0.5)^aB^	(35.4±0.7)^bA^	(32.5±1.0)^cA^
MUFA	(7.3±0.2)^aA^		(17.9±0.3)^bA^	(29.8±0.4)^cA^	(35.9±0.4)^dA^		(8.9±0.2)^aB^	(6.5±0.3)^bB^	(28.6±0.4)^cA^	(27.0±0.4)^dB^
PUFA	(41.5±0.1)^aA^		(41.5±0.3)^aA^	(24.8±0.3)^bA^	(26.1±0.4)^cA^		(42.5±0.1)^aA^	(46.7±0.3)^bB^	(28.7±0.3)^cB^	(31.8±0.3)^dB^
ω3	8.6		5.7	7.7	7.9		nd	10.8	8.2	8.6
ω6	32.9		35.8	17.1	18.2		42.5	35.9	20.5	23.2
ω3/ω6	1:4		1:6	1:2	1:2		n.d.	1:3	1:2	1:3

The same lower-case letter in a row indicates not significant differences between culture media of each strain, and the same capital letter in a row indicates significant differences between the strains in the same culture medium based on Tukey’s test (p>0.05). n.d.=not detected, SFA=saturated fatty acids, MUFA=monounsaturated fatty acids, PUFA=polyunsaturated fatty acids, ω3=omega-3 fatty acids, ω6=omega-6 fatty acids. Results are expressed as mean value±standard deviation (*N=3*)

Regardless of the used medium, palmitic (C16:0), oleic (C18:1), linoleic (C18:2) and linolenic (C18:3) acids were detected in both strains. An exception was *N. gaditana* L2 in algal medium, where linolenic acid (C18:3) was not detected. It is also notable that nutrient variation influences strongly the fatty acid composition. Higher mass fractions of SFAs and MUFAs were recorded in Conway and f/2 medium (p<0.05), whereas mass fractions of PUFAs were significantly higher in BG-11 and algal medium.

Elevated MUFA mass fractions were noticed under N-deficient conditions, and the highest MUFA accumulated is the oleic acid (C18:1) observed in *Chlorella* sp. SM1 (33.9%) in f/2 medium. Moreover, the sum of all identified fatty acids ranged from 71.5% in *N. gaditana* L2 in algal medium to the highest in *Chlorella* sp. SM1 in f/2 medium (93.5%). The overall fatty acid profiles found in this work corroborate those previously published by Mendes *et* *al.* (*51*) in *Chlorella* sp., and those reported by Jazzar *et al.* (*19*) in *Chlorella sorokoniana* and *Neochloris* sp. Fatty acid variations reported in this work were also in agreement with previous studies that suggested an increase in fatty acid content in response to nitrogen deficiency (*52*, *53*) accompanied by an increase in oleic acid content.

### Ricotta enrichment and sensory evaluation

**Fig. S2:**
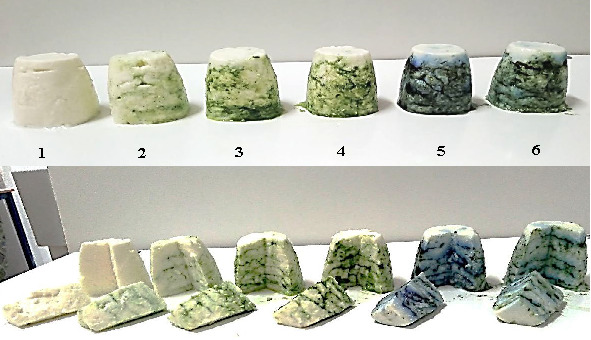
Impact of microalgal biomass pigments and mass fraction on the appearance of fresh ricotta: (1) control, (2) 0.2% *Chlorella* sp. SM1, (3) 1% *Chlorella* sp. SM1, (4) 1.5% *Chlorella* sp. SM1, (5) 1.5% commercial *Arthrospira platensis*, and (6) mixture of 1.5% *Chlorella* sp. SM1 and 1.5% *Spirulina platensis*

Making a good quality fresh ricotta cheese is still an art because of the crucial requirements for satisfactory texture and flavour. Adjusting the manufacturing procedure steps is considered critical, and even more if an additional ingredient is to be added to the original recipe. For this purpose, several assays were made at laboratory scale to adjust the manufacturing procedure and then move to small-scale assays. It was finally decided to add microalgal biomass equally between different layers of the ricotta when filling the recipients. The obtained ricotta samples with microalgal biomass had an original appearance with attractive colour in different green tonalities of the used microalgae (Fig. S2).

**Fig. 3:**
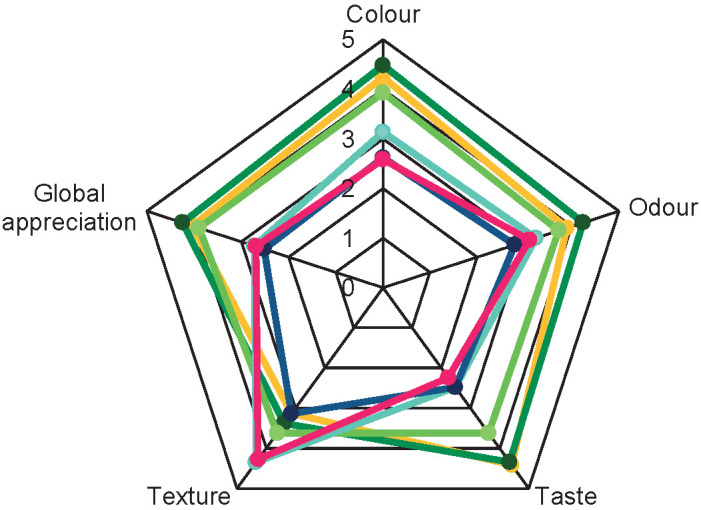
Sensory evaluation results (*N*=30) of control (yellow) and ricotta samples with 0.2 (dark green), 1 (light green) and 1.5 (turquoise) % *Chlorella* sp. SM1, 1.5% *Arthrospira platensis* (dark blue) and mixture of *Chlorella* sp. SM1 and *Arthrospira platensis* 1.5% (magenta)

The assay of ricotta cheese formulations with *Chlorella* sp. SM1 biomass obtained in BG-11 medium showed a good compromise between the biomass productivity and bioactivity results. This enrichment of cheese with *Chlorella* was compared to that of cheese prepared with a commercial *Arthrospira platensis*, which is a reference strain in food industry to better ascertain the impact of the pigment on the sensory assays. These two strains are generally recognized as safe (GRAS) and are already widely used in food formulations (*6*, *9*). Ricotta samples with mass fractions of 0.2, 1 and 1.5% *Chlorella* sp. SM1, 1.5% *A*. *platensis* and a mixture of 1.5% *Chlorella* sp. SM1 and 1.5% *A. platensis* were therefore evaluated by a sensory panel of INSAT researchers (Fig. 3).

Fig. 3 shows the average scores for the main sensory attributes (colour, odour, flavour and texture) as evaluated by the panel. The least appreciated sample was the one containing 1.5% *Spirulina platensis.* In fact, the panel showed more preference for the sample with 0.2% *Chlorella* sp. SM1 and the control one, in terms of global appreciation. Concerning the colour, the tasters classified positively the samples with microalgal biomass (0.2 and 1% *Chlorella* sp. SM1) and the 1.5% mixture as the least appreciated one.

Consumers have always been sensitive to the taste and odour when evaluating a product even before the health benefit consideration. Higher concentration of microalgae had a negative impact on the odour and flavour of the samples, which were the least appreciated.

Sensory properties of several products enriched with microalgae have already been tested and globally appreciated (*6*). However, in some cases, the addition of high mass fractions of microalgae (2%) negatively influenced the flavour parameter and led to a negative global appreciation (*13*).

In the comments section, the tasters mentioned that the ricotta with 1.5% *Arthrospira platensis* had a very unpleasant fishy odour and aftertaste. In fact, the ricotta with 0.2% *Chlorella* sp. SM1 was considered the most balanced in terms of flavour, followed by the sample with 1% *Chlorella* sp. SM1.

The texture was estimated manually by stirring the sample with a spoon. As seen in Fig. 3, the samples show a significant change in the texture with the addition of microalgae (>0.2%). This change in the texture was positively received by the panellists and reached 4 on the scale, corresponding to ’pleasant’ texture. Actually, this change could be related to the fact that standard ricotta usually has a very soft texture with a tendency to crumble very easily. Microalgal biomass brought an overall positive structural effect.

**Fig. 4:**
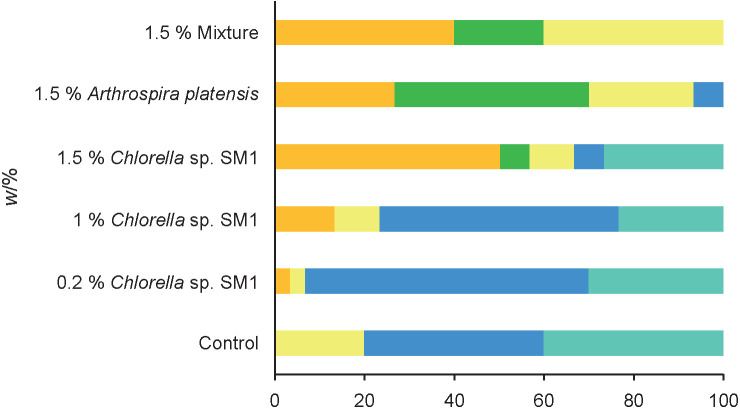
Panel tasters buying intention (*N*=30) regarding control ricotta cheese, and ricotta samples containing *Chlorella* sp. SM1 and *Arthrospira platensis*. The mixture contains 1.5% of both strains. Certainly would not buy (orange), probably would not buy (green), not sure (yellow), would probably buy (blue), would certainly buy (turquoise)

Fig. 4 gives the answers by the tasters regarding the buying intention. Sixty-three and 53% of the tasters ’would probably buy’ the ricotta with 0.2 and 1% *Chlorella* sp. SM1, respectively, while 26% of the tasters ’would certainly buy’ and 6% ’would probably buy’ the ricotta with 1.5% *Chlorella* sp. SM1. The ricotta with 1.5% *Arthrospira platensis* was the least appreciated with 26% of the tasters stating that they ’certainly would not buy’ it and 34% ’probably would not buy’. This simulation is of great importance because it helps to predict a possible future commercialization of the product since it gives a perspective of the potential consumer acceptance.

## CONCLUSIONS

This work investigated the feasibility of using microalgae to enrich and enhance the nutritional properties of ricotta, a Mediterranean dairy product made from cheese whey. This approach can be considered as a new application that allowed obtaining an innovative product. The variation of growth media confirmed the role of nitrogen-deficient medium like f/2 or Conway to boost the antioxidant activity of microalgae while keeping basal nutritional benefits. The cultivation of *Chlorella* sp. SM1 and *Nannochloropsis gaditana* L2 in f/2 and Conway media enabled high-quality PUFA accumulation. The highest antioxidant activity, carotenoid and phenolic contents were also obtained in these two media. The formulation of the enriched ricotta was successfully made without altering either the flow diagram, or the sensorial acceptability of the product, especially at low microalgal mass fraction (0.2%). The addition of microalgal biomass to fresh ricotta had a favourable impact on the sensory attributes of the final product designated ’ricottalgue’. Based on the obtained results, the attractiveness of a cheap artisanal dairy product could be enhanced using microalgae as natural and healthy ingredient.

## Figures and Tables

**Table S1:** Chemical composition of algal, BG-11, f/2 and Conway culture media

Medium component	*γ*/(g/L)			
Algal	BG-11	f/2	Conway
Macronutrient				
NO^3-^	9.9·10^-1^	1	5.5·10^-2^	7.4·10^-2^
PO_4_^3-^	8.5·10^-2^	1.7·10^-2^	3.4·10^-3^	1.6·10^-2^
Micronutrient				
Fe^2+^	7.8·10^-4^	6.5·10^-5^	2.5·10^-4^	1.9·10^-4^
Cu^2+^	7.2·10^-5^	2·10^-5^	2.5·10^-3^	5.1·10^-6^
Co^2+^	6.3·10^-5^	1.2·10^-5^	2.5·10^-3^	5·10^-6^
Zn^2+^	2.3·10^-4^	5·10^-5^	5·10^-3^	1·10^-5^
SO_4_^2-^	3.6·10^-2^	2.9·10^-2^	1.9·10^-3^	-
Mn^2+^	4.4·10^-4^	5·10^-4^	5·10^-2^	1.6·10^-4^
Mo^6+^	4.8·10^-4^	1.7·10^-4^	2.5·10^-3^	7·10^-7^
EDTA	8·10^-3^	8·10^-2^	3.4·10^-3^	3.9·10^-2^
B^3+^	-	5.1·10^-4^	-	5.9·10^-3^
Mg^2+^	-	7.4·10^-3^	-	-
Vitamin				
B1	3.9·10^-4^	-	10^-3^	2·10^-4^
B8	2·10^-5^	-	-	-
B12	1.4·10^-5^	-	5·10^-5^	10^-5^

**Table S2:** IC_50_ values of methanolic extracts of *Chlorella* sp. SM1 and *Nannochloropsis gaditana* L2 cultivated in four culture media

Medium	IC_50_/(mg/L)	
*Chlorella* sp. SM1	*Nannochloropsis gaditana* L2
Algal	(1.4±0.2)^a^	(4.26±0.02)^a^
BG-11	(1.97±0.07)^a^	(4.30±0.01)^a^
f/2	(1.2±0.3)^b^	(2.5±0.3)^b^
Conway	(1.61±0.00)^a^	(4.49±0.05)^a^
BHT	0.56±0.00	

BHT=butylated hydroxytoluene (control). Results are expressed as mean value±standard deviation (*N=3*)
